# A mixed methods pilot study with a cluster randomized control trial to evaluate the impact of a leadership intervention on guideline implementation in home care nursing

**DOI:** 10.1186/1748-5908-3-51

**Published:** 2008-12-10

**Authors:** Wendy A Gifford, Barbara Davies, Ian D Graham, Nancy Lefebre, Ann Tourangeau, Kirsten Woodend

**Affiliations:** 1 University of Ottawa, Faculty of Health Sciences, School of Nursing, 451 Smyth Road, Ottawa, ON, K1H 8M5, Canada; 2 Saint Elizabeth Health Care, 90 Allstate Parkway, Toronto, ON, Canada; 3 Canadian Institute of Health Research, 160 Elgin Street, 9^th ^Floor, Ottawa, ON, Canada; 4 University of Toronto, Faculty of Nursing, 155 College Street, Toronto, ON, Canada

## Abstract

**Background:**

Foot ulcers are a significant problem for people with diabetes. Comprehensive assessments of risk factors associated with diabetic foot ulcer are recommended in clinical guidelines to decrease complications such as prolonged healing, gangrene and amputations, and to promote effective management. However, the translation of clinical guidelines into nursing practice remains fragmented and inconsistent, and a recent homecare chart audit showed less than half the recommended risk factors for diabetic foot ulcers were assessed, and peripheral neuropathy (the most significant predictor of complications) was not assessed at all.

Strong leadership is consistently described as significant to successfully transfer guidelines into practice. Limited research exists however regarding which leadership behaviours facilitate and support implementation in nursing.

The purpose of this pilot study is to evaluate the impact of a leadership intervention in community nursing on implementing recommendations from a clinical guideline on the nursing assessment and management of diabetic foot ulcers.

**Methods:**

Two phase mixed methods design is proposed (ISRCTN 12345678). Phase I: Descriptive qualitative to understand barriers to implementing the guideline recommendations, and to inform the intervention. Phase II: Matched pair cluster randomized controlled trial (n = 4 centers) will evaluate differences in outcomes between two implementation strategies. Primary outcome: Nursing assessments of client risk factors, a composite score of 8 items based on Diabetes/Foot Ulcer guideline recommendations.

Intervention: In addition to the organization's 'usual' implementation strategy, a 12 week leadership strategy will be offered to managerial and clinical leaders consisting of: a) printed materials, b) one day interactive workshop to develop a leadership action plan tailored to barriers to support implementation; c) three post-workshop teleconferences.

**Discussion:**

This study will provide vital information on which leadership strategies are well received to facilitate and support guideline implementation. The anticipated outcomes will provide information to assist with effective management of foot ulcers for people with diabetes.

By tracking clinical outcomes associated with guideline implementation, health care administrators will be better informed to influence organizational and policy decision-making to support evidence-based quality care. Findings will be useful to inform the design of future multi-centered trials on various clinical topics to enhance knowledge translation for positive outcomes.

**Trial Registration:**

Current Control Trials ISRCTN06910890

## Background: diabetic foot ulcers

Diabetes mellitus, a complex, life-long metabolic disorder characterized by raised blood glucose concentrations, affects 4.2 percent of the world's population and over 1.5 million Canadians [1,2]. Ulceration of the foot is a significant problem for people with diabetes, affecting 15 percent at some time in their life [3,4]. Foot complications are a major reason for hospital admissions, accounting for approximately 20 percent of all diabetes-related admissions in North America [1]. Foot ulcers precede 85 percent of lower limb amputations [4,5] and 30 percent of those undergoing amputation die within the following year [6]. Diabetes pathology that increases risk of foot ulcerations and complications includes peripheral neuropathy (impairment of nerve function), peripheral vascular disease, limited joint mobility and deformity [1,4,5,7]. The triad of neuropathy, deformity, and trauma is present in almost two thirds of people with foot ulcers [5] with footwear being a major cause of traumatic ulcers [8].

Lack of awareness of risk factors associated with diabetic foot ulcer by health care professionals and patients adds to unnecessary morbidity such as prolonged healing, infections and gangrene that may result in amputations [4,5,9]. Mills et al. (1991) reviewed records of 55 diabetic patients with localized gangrene or infection on a vascular surgical unit and found 29 percent were delayed in referral for definitive care due to a lack of recognition by practitioners of ischemia or an underestimation of the severity of infections [10].

Comprehensive assessments by health care professionals of risk factors are recommended in clinical practice guidelines for effective management and treatment of diabetic foot ulcers, and are supported by strong empirical evidence [1,4-7,11-16]. A recent Cochrane review showed managing ulcers with hydrogel dressings when compared to usual care (gauze dressings) improved healing rates by 23 percent at 12 to 20 weeks (95% CI 10–36%) [7]. Assessments are recommended to include: peripheral neuropathy, vascular status, structural deformities, infection and ulcer size [1,5,9,12-15]. Referrals to multidisciplinary foot care specialists [5,12,13] and patient education [4,17] are equally emphasized.

### Problem: Implementing clinical guideline recommendations

Clinical practice guidelines synthesize and translate high quality research evidence into recommendations for practice, and provide an easy and accessible tool for bridging the evidence-practice gap [18-21]. For practice change to occur however, guidelines must be utilized, and their timely and effective transfer into clinical practice remains fragmented and inconsistent [21-24]. Implementation strategies directed at individuals, the environment and the organizational context are necessary for successful implementation and practice change to occur [20,25-27]. In recent Cochrane reviews, tailored interventions that focus on individual and organizational barriers to change showed promise for implementing change and improving patient care [28], and interactive workshops were found to have moderately large effects on changing professional practice [29].

The importance of top managers' involvement and commitment in implementing innovations such as guidelines and change have been emphasized outside [30-39] and within healthcare settings [40-45]. Descriptive and qualitative evidence has identified leadership and management behaviours as having an important impact on nurses' work environments [42,46-50] and their use of research evidence to inform practice [27,51-63]. Similarly, a systematic review of 30 studies identified the lack of support from managers, and 'other staff' to be one of the greatest barriers to nurses' use of research [60]. Management behaviours such as support and commitment [56,58,64-69], policy revisions [66,70] and monitoring of clinical outcomes [66,71] have been described as enablers to nurses' use of research [72]. Limited experimental research exists however regarding which behaviours are most effective to facilitate guideline implementation in nursing. A recent mixed methods study of 37 organizations found leadership to be the only predictor of sustained use of clinical guideline recommendations two and three years post-implementation, accounting for 47 percent of the variance (p < .001) [73]. Using grounded theory to analyze 9 of the 37 organizations, Gifford et al. found patterns of leadership and managerial behaviours in organizations that sustained practice change based on guideline recommendations (n = 4) at 2 and 3 years differed when compared to organizations that did not sustain practice change (n = 5) [63]. A conceptual model was developed from the analysis that operationalizes leadership behaviours for implementing and sustaining practice change.

### Study Aim

The aim of this pilot study is to evaluate the impact of a leadership intervention on implementing new recommendations from a clinical practice guideline on nursing assessments and management of foot ulcers for people with diabetes in community nursing practice. Specific objectives include:

1) To identify barriers and develop a tailored leadership intervention for home care nurse managers, supervisors, resource nurses and clinical staff to influence implementation of selected recommendations from the Registered Nurses Association of Ontario (RNAO) clinical practice guideline for care of foot ulcers for people with diabetes.

2) To determine the impact of the intervention on client, nurse and system outcomes.

3) To understand the feasibility of influencing leadership behaviours through the intervention.

4) To test and refine a model of leadership for implementing practice change.

We plan to test the following study hypotheses:

**H_1_**: Nurses working in centers that receive the intervention will obtain significantly higher scores for practicing in accordance with guideline recommendations than control group.

**H_0_**: No change in group means will occur following the intervention.

### Design/Methods

**Figure 1:**
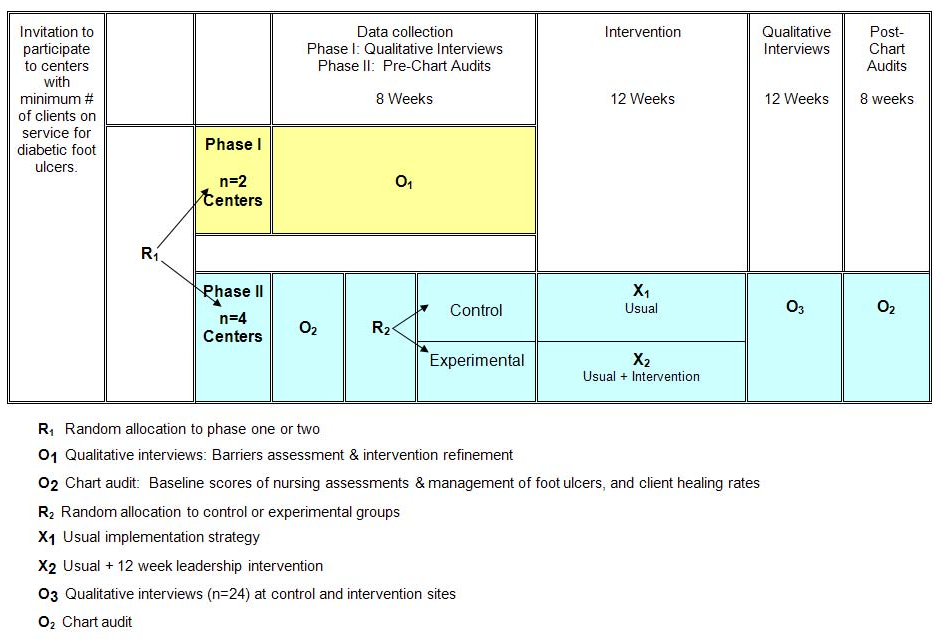
Design: Two phased mixed methods pilot study.

A two phase mixed method design is proposed (Figure 1). A pilot study is planned because there is little information regarding effective leadership behaviours for implementing practice change in nursing, and there is a need to test the intervention strategies prior to launching a larger multi-centered trial. Phase one involves descriptive qualitative methods to understand barriers to implementing the guideline recommendations and to refine the intervention strategy to be useful and appealing to leaders. A cluster randomized controlled trial, considered the optimal design when evaluating strategies to change professional behaviour [20,74], will evaluate differences in outcomes between the two implementation strategies. Randomization will occur at the unit level to minimize threats of experimental contamination [20,75,76].

#### Site

The research is being conducted in a home and community health-care service organization that provides nursing care through 23 centers in the province of Ontario Canada. The organization employs approximately 1500 nursing staff, 65 managers and supervisors, and 20 clinical resource nurses, and 7 clinical directors. Approximately 30 to 40 percent of clients receiving nursing services are diabetic, and clinical directors identified foot care for this population as a priority clinical topic, with a notable gap between current practices and guideline recommendations. For example *no *clients are currently being assessed for peripheral neuropathy the most significant predictor of ulcers, and recent chart audits indicated that co-morbidity, vascular status and wound size were not documented in at least 50 percent of charts for foot and leg ulcers. The organization has previously implemented clinical practice guidelines at an estimated cost of $60,000 per implementation. To date implementation strategies have had mixed success. Implementation of the RNAO guideline *Assessing and Managing Foot Ulcers for People with Diabetes *[13] is planned in 2008.

#### Primary outcome

Nursing assessments of client risk factors scores (NACRF), a composite score of 8 items based on recommendations from the Diabetes/Foot Ulcer guideline. The 8 items were chosen in consultation with clinical experts in diabetes and wound management, have a high level of research evidence for prediction of poor outcomes [13], and were reviewed for content validity by researchers and clinical experts in the field. Four of the eight items were previously used in a chart audit evaluation of another RNAO guideline related to the prevention of foot complications in people with diabetes [77,78].

#### Secondary outcomes

1) proportion of people with healed ulcers at 12 weeks (defined as complete wound closure),

2) healing times in number of weeks,

3) types of treatments used (eg: hydrogel dressings, sharp debridement, offloading devices),

4) referral rates to specialists services,

5) documented patient education,

6) proportion clients assessed for all items in the NACRF scale (all-or-none measure) [79],

7) Nursing participant satisfaction and perceived utility of elements of the intervention.

### Sample

All centers (approximately 10) with the minimum number of clients being treated for diabetic foot ulcers to satisfy sample size calculations will be invited to participate in the study. Two centers will be randomly assigned to participate in phase one and four will be randomly assigned for phase two. The four sites in phase two will be randomly allocated to control (n = 2) or experimental (n = 2) groups.

#### Sample size

Sample size calculations were determined, and are based on the use of an independent t-test on NACRF scores at the end of the study. The following assumptions have been made: alpha = .0.05 (two-tailed), Beta = 0.20 and an expected change in NACRF scores of 20 percent. Although all items within the NACRF have not been previously used, four were previously evaluated in a pre/post chart audit that showed a 26 percent absolute improvement in nursing documentation (range -3.6 to 57.1) [78]. Thus, an estimate of 20 percent improvement will be used. In addition, standard deviations (SD) and intra-cluster correlation coefficients (ICCs = *ρ*) for NACRF are presently unknown. It is however, estimated that the effect size may be as small as 1.00 but to be conservative 0.83 (SD = 3) is assumed for this calculation. Based on these assumptions, 30 charts will be needed in both intervention and control groups (n = 60). While it is not known exactly how many clients with diabetes will be on service for foot ulcers during the study period, senior administrators have reassured investigators that a minimum of 30 clients per group is feasible.

#### Power estimates for secondary outcomes

The anticipated rate of healing in the control group is 24 percent in 12 weeks [16]. For the proportion of ulcers healed and healing times, 30 charts in control and intervention groups would yield 80 percent power to detect an absolute increase in healing rates of 40 percent (alpha .05, two tailed). The study is also powered to detect an absolute increase of 40 percent in referral rates and patient education, also measured as a proportion.

### Data Collection

#### Baseline

All adult clients (18 years or older) diagnosed with Type 1 or Type 2 diabetes being treated for a first or recurring foot ulcer(s) will be eligible for the study. Using data abstraction forms modified from a previous guideline evaluation project [77], chart audits will be performed at control and experimental sites prior to randomization until sample size is achieved or up to 12 weeks prior to the intervention. Chart audit data collectors will be trained and supervised by researchers with experience in conducting chart audits. Interrater and test-retest reliability will be assessed in a random review of 10 percent of charts.

### PHASE I: Barriers Assessment and Intervention Development

Semi-structured interviews will be conducted at two centers with a sample of managers, supervisors, resource nurses and 2 'preceptor' staff nurses from each site (n = 10). Preceptor staff are experienced clinical nurses who volunteer to provide support to novice or newly hired nurses regarding clinical issues. The interview guide is based on previously published guides for assessing barriers and supports [80], and has been structured to understand components of an intervention strategy considered useful to managers and clinical leaders. Results of phase I will inform content and structure of the intervention strategy.

### PHASE II: Intervention Strategy

#### Control Group

Staff at each center will receive the 'usual' guideline implementation strategy consisting of: 1) a formal guideline launch; 2) self-directed learning package, 3) educational sessions for staff related to the clinical application of practice recommendations. Senior administrators estimated that approximately 70 percent of staff typically attend 'usual' strategies.

#### Experimental Group

In addition to the 'usual' implementation strategy, a 12 week leadership strategy will be offered to mangers, supervisors, resource nurses, and 2 preceptor staff from each center to facilitate and support implementation, consisting of:

1) Mailed package of printed materials: to include study purpose; summary of recommendations, models of leadership and planned change; literature article; three questions to assess barriers to nurses assessing and managing foot ulcers in accordance to the guideline recommendations. Review time: approx 15–30 minutes.

2) Interactive workshop (one day): Content and activities will be tailored to results of phase one, planned to include: a) evidence and theory on leadership and implementing practice change; b) focus group discussions about barriers to implementing the recommendations; c) role playing exercises; and d) facilitated development of a team leadership implementation plan for each center, tailored to identified barriers.

3) Post-workshop teleconferences: (2, 6, and 10 weeks after workshop) to provide a forum for questions, discussions and networking amongst participants.

### Guiding Theoretical Framework

The theoretical underpinnings of the proposed intervention are based on mechanisms of planned change as described in the Ottawa Model of Research Use (OMRU^©^) [52,81], effective leadership behaviours described by Yukl [82], and leadership for guideline implementation described by Gifford et al [63].

The OMRU is a planned change framework for knowledge transfer in health care delivery [52]. Derived from evidence and theories of change, the OMRU recognizes that practice change is not a linear process, but involves simultaneous and interactive relationships between the nature of the innovation, the potential adopters, and the context within the practice environment. Three key processes involved are: 1) assessing barriers and supports; 2) developing and monitoring interventions tailored to barriers and supports; 3) evaluating outcomes. The underlying mechanism is that tailoring intervention strategies to address barriers and strengthen supports related to the innovation, potential adopters and practice environment will result in practice change.

The OMRU provides a template to assess barriers and supports for implementing change and will facilitate the selection of intervention strategies with the best probability of success. The relevance and pragmatic utility of the OMRU for guiding implementation of innovations (including nursing guidelines) has been demonstrated in previous research [83-87].

Leadership is "the process of influencing others to understand and agree about what needs to be done and how to do it, and the process of facilitating individual and collective efforts to accomplish shared objectives" [[82], p.8]. Three meta-categories of effective leadership behaviours described by Yukl and supported by decades of research [82,82,88,89], provide the foundation for this study: 1) relations-orientated, 2) change-orientated and 3) task-orientated. Relations-oriented behaviours include supporting, developing personal skills and job adjustments, and recognizing others and their contributions. Relations-oriented behaviours increase mutual trust, cooperation among members, and commitment to a unit and organization. Change-oriented behaviours are concerned with integrating a vision, developing strategies and building coalitions to support change, creating a sense of need and demonstrating commitment to change. Task-oriented behaviours include clarifying roles, monitoring operations and performance, and the efficient use of resources [82].

Three leadership themes emerged as central to implementing guidelines in the grounded theory study by Gifford et al., and these align closely with Yukl's [82] metacategories of effective leadership behaviours. Leaders were found to have: 1) facilitated staff through relations-oriented behaviours (e.g.: support, encouragement and recognition); 2) created a positive milieu within the clinical practice environment through change-related behaviours (e.g.: reinforced goals and philosophies of care); and 3) influenced organizational structures and processes through task-oriented behaviours (e.g.: providing resources, policies and monitoring). Together these behaviours influenced individuals, practice environments and infrastructures to enable nurses to practice based on guideline recommendations.

**Figure 2:**
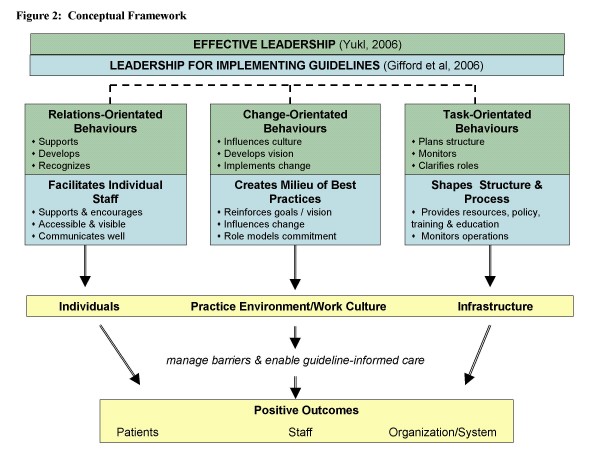
Conceptual Framework.

Drawing on the work of Van de Ven et al. (1999), effective leadership at different hierarchical levels is necessary for the adoption of new innovations in organizations [90]. Successful implementation in healthcare is dependent on strong effective leadership to create a context which is receptive to change [26,27,51,63,82,90-96]. The organizational context exerts a particularly powerful set of influences on nurses' adoption of new innovations [81,97,98]. Extensive managerial involvement, commitment and attitude toward change, role clarity, and leadership styles are significantly associated with maintaining the momentum of innovation adoption in organizations [32,33,90,99,100]. A 'road map" that explains what leaders do is not however possible due to the inherent unpredictability and nonlinear processes of innovation adoption [90]. "Management cannot ensure innovation success but can influence its odds" (p.11, 88). Leadership is an integral part of managerial roles, and is necessary for managers to influence change [34,82,96,101-104]. Individuals and organizational context must be influenced for practice change to occur based on new innovations [20]. The proposed intervention aims to influence individuals, the practice environment and organizational context through leadership processes and behaviours that manage barriers and enable practice change to occur. (Figure 2)

### Post-intervention measures

Chart audits will be conducted on all patients being treated for diabetic foot ulcers up to 12 weeks following the intervention. To understand the leadership and management behaviours that influenced nursing practice, semi structured qualitative interviews will be conducted with managers, supervisors and resource nurses and staff nurses at control and experimental sites (n = 20). The experimental group interview guide will also ask for participants' opinions regarding the usefulness of the intervention. The interview guides are based on previously published guides for assessing barriers and supports [80], and previous research on implementing guidelines [105]. To evaluate satisfaction and perceived utility of the one day workshop, an evaluation form, based on previously evaluations from RNAO guideline implementations, [106] will be administered at the end of the workshop.

### Data Analysis

Pre/post univariate descriptive data will be computed for demographics of patients and staff.

#### Primary Outcome: Composite NACRF scores

Eeach item within the scale will be coded dichotomously (1 = yes; 0 = no), and a total score calculated out of 8. Bivariate analysis using independent groups t-tests will be conducted to assess the significance of differences pre/post intervention between control and experimental groups. The alpha level will be pre-set at .05, and 95 percent confidence intervals calculated. An 'intent to treat' analysis will be used [75].

#### Secondary Outcomes

The proportion of people with healed ulcer(s) at 12 weeks, and time to complete healing will be calculated. Types of treatments used (eg: hydrogel dressings, sharp debridement, offloading devices) will be calculated. Clients with documented patient education and referrals will be dichotomously coded (1 = yes; 0 = no/don't know). Independent groups t-tests for continuous variables, and chi squares for categorical variables will determine differences before and after the intervention within each center, and between control and experimental groups. Descriptive statistics will be used to evaluate nursing participants' satisfaction and perceived utility with the elements of the intervention.

#### Other Outcomes

ICCs (*ρ*) will be calculated on pre/post measures of composite NACRF scores, and demographic characteristics of clients (e.g.: age, gender) [107]. Matching is expected to minimize between-unit variations, and previous research shows ICCs for the process of care to be high [20,74,108]. ICCs from this study will be useful to inform future studies regarding sample size calculations [107,109,110].

#### Qualitative Findings

To understand how the intervention influenced leadership practices, data from qualitative interviews will be audio-taped, transcribed, entered into qualitative software (NVIVO) and analyzed using content analysis techniques involving an iterative process of data reduction, data display, conclusion drawing and verification [111].

## Discussion

### Limitations

An inherent limitation of collecting data through chart audit is the documented data obtained may potentially underestimate actual care [112]. Other methods of data collection, such as direct observations are not feasible for this pilot study due to geographical distances and associated costs of observing home-care nurses provide care in patients' homes throughout the province. A second limitation of collecting data through chart audits involves reviewers accuracy, impartiality, attentiveness and consistency in extracting data [112]. Having an experienced research manager overseeing the process, and pilot testing for interrater and test-retest reliability will assist with addressing this limitation. Additionally, this is a pilot study and not sufficiently powered to account for the effect of clustering.

### Ethical Considerations

Prior to commencement, ethical approval will be obtained from University of Ottawa Research Ethics Board which follows Tri-council guidelines [113]. Details of ethical considerations, including informed consent, anonymity and confidentiality are found in ethics submission form. Briefly, a numerical coding system will be used to track individual participant and chart audit data. Names of interview participants will be kept separated from data collection forms and locked at the University of Ottawa Nursing Best Practice Research Unit. Names from chart audits will be kept by the research manager at the participating organization in a secured place; only numerically coded data will be sent to investigators. Only aggregated data will be reported. Information consent forms will be available in English and French. Data will be securely stored for 5 years after study conclusion (e.g. December, 2014).

### Feasibility

This study aligns with the participating organization's timeline to implement the *Diabetes/Foot Ulcer BPG*, and has been developed in consultations with senior administrators to ensure feasibility, support, and compatibility with organizational direction, initiatives and training strategies.

#### Potential Impact on Nursing Care

This pilot study will contribute to the development of leadership strategies to facilitate implementation of guideline recommendations on a priority clinical topic in community nursing. The anticipated outcome is information to assist with more effective management and faster healing of foot ulcers in community health nursing for people with diabetes. With the high cost of guideline implementation, this study will provide vital information on which strategies are well received when implementing practice change. By tracking clinical outcomes associated with guideline use, nursing administrators will be better informed to influence organizational and policy decisions to support high quality nursing care. Findings will be useful to inform the design of future multi-centered trials on various clinical topics, and to enhance the science of knowledge translation for evidence-informed practice change that impacts quality nursing care and client outcomes.

## Competing interests

The authors declare that they have no competing interests.

## Authors' contributions

WG and BD conceptualized the study. WG led the writing and application for funding. All other authors contributed to conceptualizing based on specific areas expertise: IG for knowledge translation framework and tool development; NL for organizational feasibility and data collection methods; AT for leadership development theory and leadership outcomes; KW for quantitative methodology and power analysis. All authors have read drafted versions of the manuscript, provided input and refinements, and agreed to the final manuscript.
